# A novel nonsense mutation of *ERCC2* in a Vietnamese family with xeroderma pigmentosum syndrome group D

**DOI:** 10.1038/s41439-020-0089-z

**Published:** 2020-02-10

**Authors:** Chi-Bao Bui, Thao Thi Phuong Duong, Vien The Tran, Thuy Thanh T. Pham, Tung Vu, Gia Cac Chau, Thanh-Niem Van Vo, Vinh Nguyen, Dieu-Thuong Thi Trinh, Minh Van Hoang

**Affiliations:** 1 Biomedical Research Center, School of Medicine, Vietnam National University, Ho Chi Minh City, Vietnam; 2 Functional Genomics Unit, DNA Medical Technology, Ho Chi Minh City, Vietnam; 3 Department of Dermatology, University of Medicine and Pharmacy at Ho Chi Minh City, Ho Chi Minh City, Vietnam; 4 Department of Molecular Cell Biology, Samsung Biomedical Research Institute, Sungkyunkwan University School of Medicine, Suwon, Gyeonggi-do Korea; 5 Center for Molecular Biomedicine, University of Medicine and Pharmacy at Ho Chi Minh City, Ho Chi Minh City, Vietnam; 6 Graduate School of Medicine, Osaka University, Osaka, Japan; 7 Department of Hematology and Dermatology, University Medical Center 3, Ho Chi Minh City, Vietnam; 8 Faculty of Traditional Medicine, University of Medicine and Pharmacy at Ho Chi Minh City, Ho Chi Minh City, Vietnam

**Keywords:** Genetics research, Genetic predisposition to disease

## Abstract

Xeroderma pigmentosum (XP) group D, a severe disease often typified by extreme sun sensitivity, can be caused by *ERCC2* mutations. *ERCC2* encodes an adenosine triphosphate (ATP)-dependent DNA helicase, namely XP group D protein (XPD). The XPD, one of ten subunits of the transcription factor TFIIH, plays a critical role in the nucleotide-excision repair (NER) pathway. Mutations in XPD that affect the NER pathway can lead to neurological degeneration and skin cancer, which are the most common causes of death in XP patients. Here, we present detailed phenotypic information on a Vietnamese family in which four members were affected by XP with extreme sun sensitivity. Genomic analysis revealed a compound heterozygous mutation of *ERCC2* that affected family members and single heterozygous mutations in unaffected family members. We identified a novel, nonsense mutation in one allele of *ERCC2* (c.1354C > T, p.Q452X) and a known missense mutation in the other allele (c.2048G > A, p.R683Q). Fibroblasts isolated from the compound heterozygous subject also failed to recover from UV-driven DNA damage, thus recapitulating aspects of XP syndrome in vitro. We describe a novel *ERCC2* variant that leads to the breakdown of the NER pathway across generations of a family presenting with severe XP.

## Xeroderma pigmentosum: New mutation linked to sun-sensitivity disorder

A novel mutation in a gene responsible for repairing DNA helps explain why members of one Vietnamese family suffer from heightened sensitivity to ultraviolet rays from sunlight. A team led by Minh Van Hoang from the University Medical Center, Ho Chi Minh City, Vietnam, sequenced the protein-coding portions of the genomes from one brother and two sisters affected by xeroderma pigmentosum, an inherited disorder characterized by extreme sun sensitivity in the skin and eyes. The researchers found that all three siblings carried two different mutations, one in each copy of a DNA-repair gene called *ERCC2*. One mutation had previously been implicated in many cases of xeroderma pigmentosum, but the other had never been reported before. These findings could aid in the diagnosis, prevention and treatment of this rare hereditary condition.

## Introduction

Xeroderma pigmentosum (XP) syndrome, which is inherited in an autosomal recessive manner, is characterized by sun sensitivity (severe sunburn with irritation and blistering, dry skin, freckles, hyper- and hypopigmentation) and sunlight-induced ocular symptoms (photophobia, photosensitivity, impaired vision, and cataract)^1^. Approximately 25% of XP patients have neurological abnormalities, including cognitive decline, loss of reflex and tonus, progressive hearing loss, and ataxia. The average life expectancy of XP individuals with and without neurological degeneration is 29 and 37 years old, respectively^1^.

XP diagnosis requires both clinical examination and genetic analysis. Sethi et al.^2^ reported that while sun sensitivity in XP correlated with disease severity scoring, half of all patients had no history of abnormal sunburn. Genetic analysis has revealed a potential mechanism for this type of sun sensitivity in selected XP patients through variants in nucleotide-excision repair (NER) genes. NER pathway genes, including *DDB2*, *ERCC1*, *ERCC2*, *ERCC3*, *ERCC4*, *ERCC5*, *POLH*, *XPA*, and *XPC*, eliminate DNA damage by UV light or chemical carcinogens^1^. In particular, *ERCC2* (OMIM: 126340) encodes the XP group D protein (XPD), a DNA helicase and subunit of transcription factor TFIIH, which is involved in basal transcription and NER. Thus, mutations in XPD that disrupt NER can lead to XP.

A number of mutations in *ERCC2* have previously been reported to cause XP group D (OMIM: 278730), trichothiodystrophy 1 (OMIM: 601675), and cerebrooculofacioskeletal syndrome 2 (OMIM: 610756)^3,4^. Notably, a 4-bp deletion at nucleotides 668–671 and a S541 mutation in *ERCC2* were reported in a Japanese XP patient^5^. Another study described a -2 frame shift mutation with a loss of two Ts at nucleotides 1781–1782 on one allele, a deletion of nucleotides 1823–1825 (AGA), and an insertion of TTTCGG at this site on the other allele^6^. The same study also described another patient with R112H and L485P mutations^6^. A further mutation of R616Q has been reported in one individual, albeit presenting with a milder sun sensitivity^7^. However, the most common mutations found in XP patients are the R683Q/W variants^7–11^.

Herein, we describe three siblings with extreme sun sensitivity who carry the p.R683Q missense mutation in one allele but also have a novel p.Q452X nonsense mutation in the other allele of the *ERCC2* gene. These patients, captured within a genetic analysis across three generations of the same family, provide further insights into the molecular basis of XP.

## Materials and methods

### Phenotypes of affected individuals

Three patients (II-4, II-6, and II-10) were first recruited to the Medical University Center 3 in 2013. These patients were all siblings of a family living in Tay Ninh Province, Vietnam. Physicians recorded a detailed phenotypic analysis of each patient to include dermatology, ophthalmology, and neurology pathologies based on the XP Disease Severity Scoring System^2^. Family members provided individual, written informed consent for undergoing a clinical assessment and detailed genetic analysis and being photographed for publication purposes under the University Medical Center ethics board (15 people, 3 generations). Informed consent was not sought for those individuals who were deceased (four people).

### Whole-exome sequencing (WES)

Genomic DNA was extracted from peripheral blood using the QIAamp DNA Blood Mini Kit (#51104, QIAGEN, Venlo, LI, the Netherlands). DNA was examined for quality using DropSense96, Qubit 2.0, and TapeStation. The prepared libraries were sequenced by 2 × 100 bp paired-end sequencing using Agilent SureSelect Human All Exon V5 (Agilent Technologies, Santa Clara, CA, USA) on a NovaSeq 6000 Sequencing System (Illumina, San Diego, CA, USA) by Macrogen company in Seoul, Republic of Korea. Using paired FASTQ reads, FASTQC was used to obtain useful diagnostic scores, including the Phred-score distribution along the reads, GC content distribution, read-length distribution, and sequence duplication level. Subsequently, trimmomatic was used to remove low-quality bases and adapter sequences. Preprocessed read pairs were mapped to GRCh37/hg19 (UCSC) by BWA-MEM. Additional processing included MarkDuplicates by PICARD and Base Quality Score Recalibration by GATK. VCF files were generated with GATK Haplotype Caller and filtered by GATK Variant Filtration, SNP (QD < 2.0, FS > 60.0, MQ < 40.0, MQ RankSum < −12.5, Read Pos Rank-Sum < −8.0) and INDEL (QD < 2.0, FS > 200.0, Read Pos Rank-Sum < −20.0). Finally, ANNOVAR was used to intersect variant annotations from UCSC RefSeq, dbSNP 150, gnomAD, ESP6500, ExAC, 1000G, and dbNSFP v3.5.

For two of the patients, II-4 and II-6, we generated 28,864,756 and 24,816,804 raw reads; 97% and 95.9% reads mapped to the reference genome with at least 10× coverage after preprocessing; average depths of 59× (96% above 10×) and 66× (95% above 10×) on captured exome intervals, respectively.

### Variant prioritization and Sanger sequencing

We applied an in-house bioinformatics pipeline for the WES analysis. Rare variants with MAF < 0.01 in several population databases were selected for further analysis. dbNSFP was utilized to identify the top candidate variants by compiling prediction scores from 29 algorithms (SIFT, SIFT4G, Polyphen2-HDIV, Polyphen2-HVAR, LRT, MutationTaster2, MutationAssessor, FATHMM, MetaSVM, MetaLR, CADD, VEST4, PROVEAN, FATHMM-MKL coding, FATHMM-XF coding, fitCons, LINSIGHT, DANN, GenoCanyon, Eigen, Eigen-PC, M-CAP, REVEL, MutPred, MVP, MPC, PrimateAI, GEOGEN2, and ALoFT) and 9 conservation scores (PhyloP × 3, phastCons × 3, GERP++, SiPhy and bStatistic). The family segregation of all detected variants was validated by Sanger sequencing.

### Unscheduled DNA synthesis (UDS) assay

Primary dermal fibroblast cells were isolated from patients and normal individuals following an established protocol^12^. Cells were then seeded on 96-well microtiter plates and cultured for 16 h in FBS-supplemented DMEM. Half of the plates were irradiated with UVC (UV+), whereas the other half were wrapped in aluminum foil (UV–). Cells were then incubated in serum-free EdU (Invitrogen, Waltham, MA, USA) supplemented with DMEM immediately after the irradiation per the established protocol^13^. Cells were then incorporated with EdU, fixed in 4% paraformaldehyde, and treated with Alexa Fluor 488-azide coupling solution (Invitrogen, Waltham, MA, USA). Nuclei were stained with DAPI (Thermo Fisher Scientific, Waltham, MA, USA). The fluorescence intensity of Alexa Fluor 488 and DAPI in 25 different fields was calculated for each cell type.

### Cell viability assay

Cell viability was examined by the alamarBlue® assay (Thermo Fisher Scientific, Waltham, MA, USA). Briefly, cells were plated at a concentration of 10,000 cells per well in a 96-well plate and then laid out according to UV irradiation conditions measured 24 h post UVR. Fluorescence intensity was measured using an excitation wavelength of 540–570 nm (peak excitation was 570 nm) and emission wavelength of 580–610 nm (peak emission was 585 nm) with a Spectra Max 384 Plus Spectrophotometer (Molecular Devices, San Jose, CA, USA).

## Results

### Clinical manifestations

**Fig. 1:**
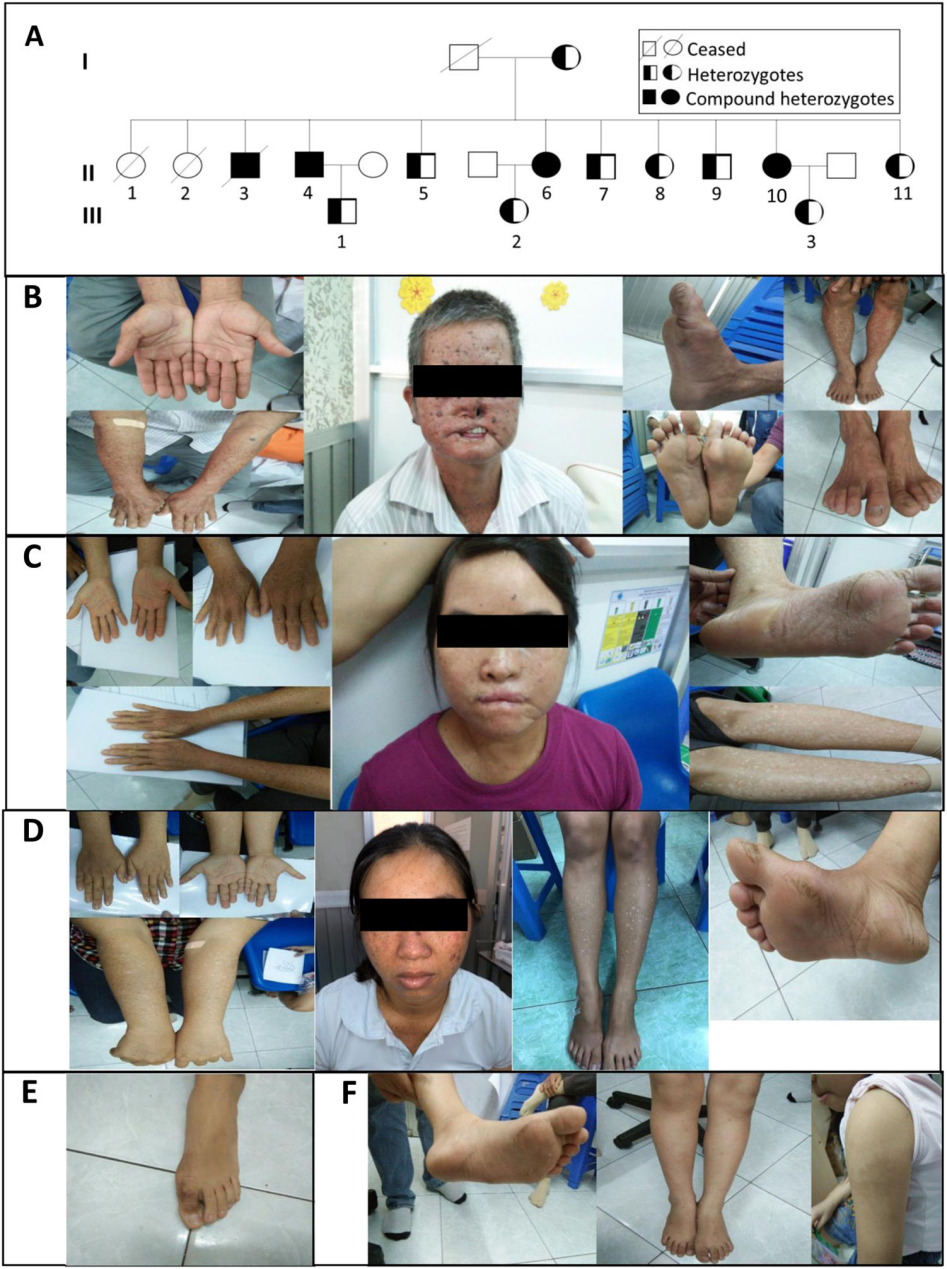
Clinical findings of patients with XP. **a** The pedigree of the 3-generation family. Circles represent females; squares represent males. Solid symbols denote individuals who are compound heterozygotes (c.1354C > T, p.Q452X) and (c.2048G > A, p.R683Q) in *ERCC2*. Half-solid symbols denote heterozygote carriers of *ERCC2*. Open symbols denote unaffected individuals. Each generation of the family is denoted as I, II, and III, while each individual of each generation is denoted as 1, 2, 3, etc. **b** Photographs illustrate severe XP in patient II-4. **c** Photographs illustrate severe XP in patient II-6. **d** Photographs illustrate severe XP in patient II-10. **e** Photographs illustrate suspicious skin conditions in subject III-2. **f** Photographs illustrate suspicious skin conditions in subject III-3.

We identified four siblings (II-3, II-4, II-6, and II-10) affected by XP in a 3-generation family, as shown in the pedigree analysis in Fig. 1a. The mother is still alive, while the father died at age 72 with no evidence of XP. Their first child (II-1) died at age 12, and their second child (II-2) died at age 14; however, the cause of death for them was not disclosed. The third child (II-3) died at age 40 and had been diagnosed with XP. The parents and other siblings have no significant family history of sun sensitivity, skin tumors, or other medical problems, as summarized in Table 1, except for patient II-5, who showed some symptoms of mild XP.

**Table 1:** Summary of the clinical examinations of XP patients.

ID	II-3	II-4	II-6	II-10	II-5	II-7	II-8	II-9	II-11	III-1	III-2	III-3
Age at onset	2 y	2 y	2 y	2 y								
Age at diagnosis	39 y	38 y	35 y	28 y	37 y	34 y	33 y	30 y	27 y	9 y	7 y	5 y
Sex	Male	Male	Female	Female	male	male	female	male	female	male	female	female
Ocular symptoms												
Photophobia	+	+	+	+	+	+	−	−	+	+	+	+
Dry eye	+	+	+	+	+	−	−	−	−	−	−	−
Impaired vision	NA	+++	−	−	−	−	−	−	−	−	−	−
Photosensitivity	+	+	+	+	−	−	−	−	−	−	−	−
Cataract	NA	+	−	−	−	−	−	−	−	−	−	−
Myopia	−	−	−	−	−	−	−	−	−	−	−	−
Retinal neovascularization	−	−	−	−	−	−	−	−	−	−	−	−
Neurologic phenotype												
Cognitive decline	−	−	−	−	−	−	−	−	−	−	−	−
Loss of tendon Reflexes	−	−	−	−	−	−	−	−	−	−	−	−
Loss of tonus	−	−	−	−	−	−	−	−	−	−	−	−
Hearing loss	+	+	−	−	−	−	−	−	−	−	−	−
Ataxia	−	−	−	−	−	−	−	−	−	−	−	−
Stroke	+++	+++	−	−	−	−	−	−	−	−	−	−
Responses to sun exposure												
Sunburn	+++	+++	+++	+++	−	+	−	−	−	−	−	−
Irritations	+++	+++	+++	++	−	+	−	−	−	−	−	−
Blisters	−	−	−	−	−	−	−	−	−	−	−	−
Freckles	+++	+++	+++	+++	−	+	−	−	−	−	−	−
Skin peeling	++	++	++	+	+	+	−	−	−	−	−	−
Hyperpigmentation	+++	+++	++	++	−	−	−	−	−	−	−	−
Hypopigmentation	+	++	++	++	−	−	−	−	−	−	−	−
dry skin	+++	+++	+++	+++	+	+	+	+	−	−	−	−
Tumor	NA	+	+	−	−	−	−	−	−	−	−	−
Type of tumor	NA	Basal Cell Carcinoma, Melanoma	Melanoma									

Explanation of symbols: (−): not present; (+): mild; (++): moderate; (+++): severe; (NA): not applicable.

Patients II-3, II-4, II-6, and II-10 (two males and two females) developed severe sunburn and irritation after short, unprotected sun exposure at approximately age 2 years (Table 1). One male patient (II-3) was deceased and thus was not available for further clinical observations; however, information was obtained through other family members. Photographs of patients II-4, II-6, and II-10 are shown in Fig. 1. The sun-exposed skin of the face, neck, shoulders, arms and legs of patients II-4, II-6, and II-10 had numerous freckle-like macules and papules (Fig. 1b–h, i–n, o–t, respectively, and Table 1). The skin was extremely dry, flaky and rough, particularly on the legs, with evidence of hyperpigmentation. Hypopigmentation, a symptom characterized as an area of skin becoming lighter than the baseline skin color, also developed on the legs of all patients (Fig. 1e–h, m–n, s–t, respectively, and Table 1). Skin peeling, a symptom characterized as a loss of the upper layer of skin, appeared more severe in patients II-3, II-4, and II-6 than in patient II-10 (Fig. 1 and Table 1). All patients had a history of photophobia, photosensitivity and dry eye upon sun exposure. Patient II-4 had progressive ocular damage leading to severely impaired vision and cataracts (Table 1). Neither developmental disabilities nor brittle hair was found in these patients.

Patient II-4 had undergone several surgeries for the removal of basal cell carcinoma and melanoma. Notably, he had a nasal tumor at age 9 that required significant resection of much of the nose (Fig. 1b). Patient II-6 also had several basal carcinomas removed by surgery (Table 1). Patient II-10 had not yet developed any malignancies (Table 1). It is worth noting that patients II-6 and II-10 had been carefully protected from sun exposure in comparison with their affected brother, II-4. Patients II-06 and II-10 had not yet developed neurological abnormalities; however, patient II-04 had a loss of hearing and previously suffered a stroke, leaving him bedridden. Information provided by the family members indicated that patient II-3 also had a stroke before his death (Table 1).

Patients II-4, II-6, and II-10 had the most, second and least extreme XP symptoms and were diagnosed at ages 38, 35 and 28, respectively. Genetic analyses were carried out to confirm the diagnosis of XP.

Interestingly, a son of patient II-4 (III-1) had no skin abnormalities, while a daughter of patient II-6 (III-2) and a daughter of patient II-10 (III-3) revealed several brownish flat macules on their arms, feet and legs (Fig. 1u, v–x). The two daughters (III-2 and III-3) were followed for further symptoms, and their blood was collected for genetic analysis.

### Genetic analysis

**Fig. 2:**
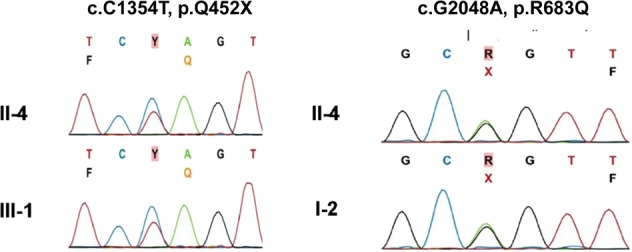
Sanger sequencing of selected family members. Patient II-4 has a compound heterozygote mutation (c.1354C > T, p.Q452X) and (c.2048G > A, p.R683Q) in *ERCC2*. The offspring of patient II-4, subject III-1, has a single heterozygous mutation (c.1354C > T, p.Q452X). Subject I-2, the mother of patient II-4, has a single heterozygous mutation (c.2048G > A, p.R683Q).

Three patients, including one male and two females (patients II-4, II-6, and II-10, pedigree in Fig. 1a), underwent WES to identify the causative genes of XP. The whole-exome data further revealed a compound heterozygote, including a nonsense mutation in one allele (c.1354C > T, p.Q452X), which has not been seen in the gnomAD or ExAC database. A missense mutation was found in the other allele (c.2048G > A, p.R683Q) of *ERCC2*, which has been seen two times in the gnomAD database as a heterozygous mutation and has not been seen in the ExAC database. The latter mutation was first reported in 2012^7^, while the former is a novel mutation to this study. Sanger sequencing verified that the heterozygous (c.2048G > A, p.R683Q) is presented by the mother (I-2), while (c.1354C > T, p.Q452X) is segregated to III-1 (Fig. 2). The parents and other siblings were heterozygotes and unaffected by XP; thus, the father (I-1) must also be considered a heterozygote. In addition, the third generation offspring (III-1, III-2, and III-3) were also heterozygotes and unaffected by XP, implying that *ERCC2* has an autosomal recessive mode of inheritance in familial cases.

### Response of fibroblasts to UV irradiation

**Fig. 3:**
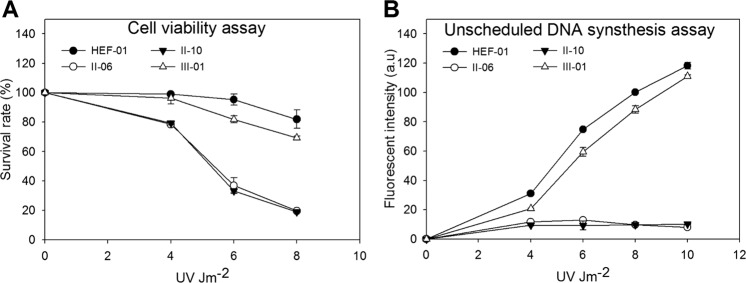
Cell viability and UDS assays. **a** Cell viability of fibroblasts measured as the survival rate following UV treatment of patients II-06, II-10, and III-01 with different UV doses (2, 4, 6, and 8 Jm^−2^) compared with the no mutation/healthy fibroblasts, HEF-01. The survival curves of patients II-06, II-10, and III-01 and HEF-01 cells are depicted. **b** UDS was measured as the fluorescence intensity following UV treatment of patients II-06, II-10, and III-01 with different UV doses (2, 4, 6, 8, and 10 Jm^−2^) and was compared with that of the no mutation/healthy fibroblasts, HEF-01. The UDS curves of patients II-06, II-10, and III-01 and HEF-01 cells are depicted.

The cell viability of skin fibroblasts from XP in single mutation/unaffected carriers and no mutations/healthy subjects after UV treatment at various doses showed that cells from XP patients (II-06 and II-10) were more susceptible to damage than unaffected and healthy subjects (III-01 and HEF-01) (Fig. 3a). UDS in skin fibroblasts from patients II-06 and II-10 was approximately 5% of that in a normal subject after treatment at a UV dose of 10 Jm^−2^. This implies that fibroblasts from the compound heterozygous patients were more sensitive to UV irradiation than wild-type cells were (Fig. 3b). UDS in the skin fibroblasts of subject III-01 was approximately 95% of that of a healthy subject after treatment at a UV dose of 10 Jm^−2^. This result was consistent with subject III-01 who carries a single heterozygous mutation but is unaffected by XP (Fig. 3b).

## Discussion

In this study, we reported a 3-generation family in which four members were affected by XP, some having presented previously with skin cancer and suffering neurological abnormalities. Genetic analyses determined these patients to be compound heterozygotes with two different mutations in the *ERCC2 gene*; one of these was a novel nonsense mutation. Consistent with genetic analyses, in vitro experiments using fibroblasts from XP-affected family members demonstrated a defect in the NER pathway.

Most previously reported XP patients, including ours, have mutations at residue R683 containing two substitutions of R683Q^7^ and R683W in XPD^8–11^. Residue R683 is involved in interacting with TFIIH and binding to double-stranded/single-stranded DNA junctions. The R residue replaced with a W or Q changes the positive charge at this position, leading to diminished DNA binding and resulting in the XP disease phenotype^14^. Falik-Zaccai et al.^7^ reported an 18-year-old patient who carried compound heterozygous mutations (i.e., p.R683Q in one allele and p.R616Q in the other allele of the *ERCC2* gene) but had a mild sun sensitivity, no skin cancer and no neurological abnormalities. They also reported four patients who were homozygotes of p.R683Q and developed a mild sun sensitivity^7^. Our finding of an additional, novel null mutant in compound heterozygous patients could reveal the cause of their extreme phenotype likely because a stop at codon 452 generates a truncated and potentially nonfunctional XPD.

**Fig. 4:**
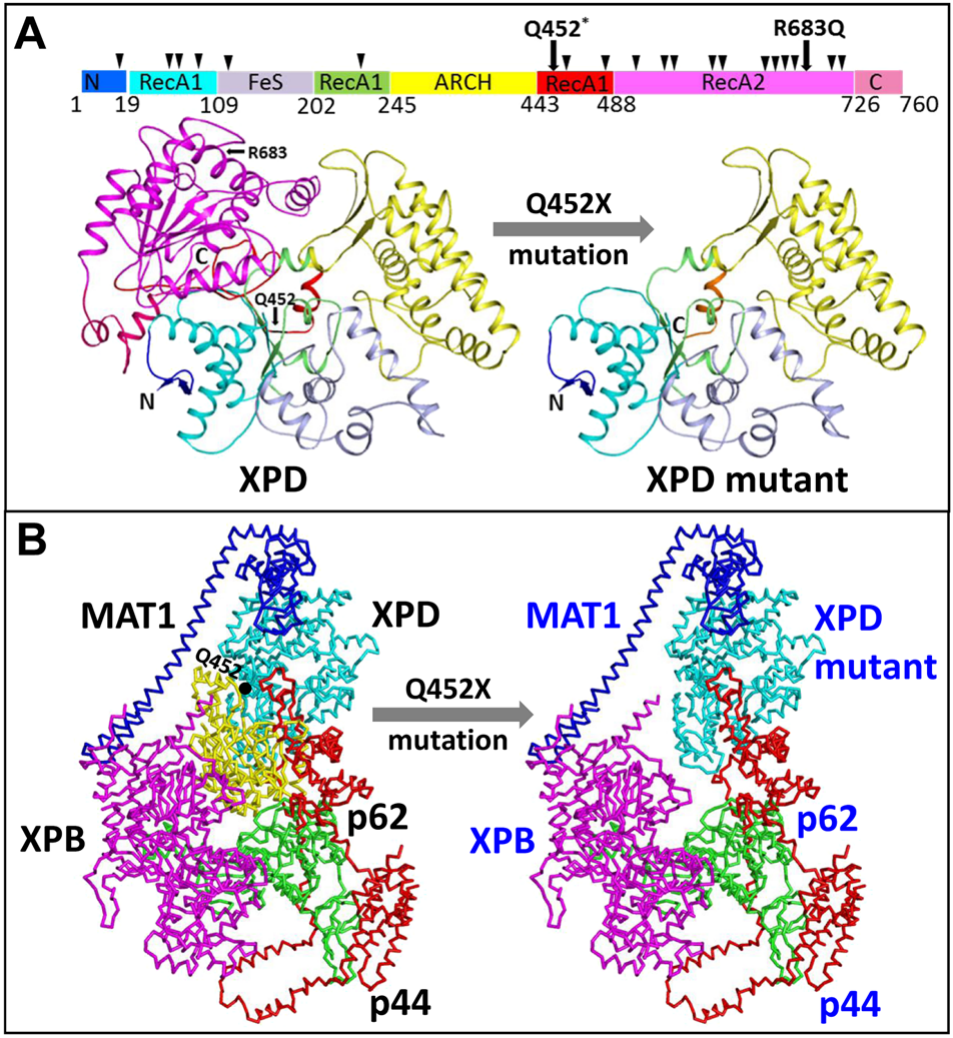
**a** Domain organization and structures of the XPD protein and Q452X-XPD mutant. Reported XP mutations are shown as a triangle above the domain map. The overall structures of XPD (left) and Q452X-XPD mutant (right) are shown in a schematic form. The N- and C-termini and the point mutations are labeled. The domains of XPD and the Q452X-XPD mutant are coded following that in the domain map. **b** Interaction network of XPD with the surrounding TFIIH subunits. The ribbon models of MAT1, XPB, p62, and p44 are depicted. The Q452 residue and residues 1–451 and 452–760 of XPD are labeled. The structures were created using PyMOL (http://www.pymol.org).

XP is one of a number of diseases associated with defects in the NER pathway, which is a pathway that involves DNA damage recognition followed by lesion verification. The proofreading step to verify DNA damage in eukaryotes is carried out by the TFIIH complex, in which XPD has helicase activity to unwind DNA and identify the DNA lesion. To predict the potential structural consequences of the Q452X-XPD mutant, we searched for the wild-type structure of XPD in the Protein Data Bank (PDB). Mining revealed several crystal structures of XPDs from different species and some identified mutations causing XP disease in humans^14^. The cryo-EM structure of the human TFIIH core complex at 3.7 Å and the refined coordinate model (PDB ID: 6NMI) have just been published, and the interaction of XPD with other subunit proteins has been characterized^10,11^. XPD contains two RecA-like domains in which the RecA1 domain is inserted by the FeS and ARCH domains, while the RecA2 domain is still intact. When mapping mutations of XP disease on the structure of XPD, most of them cluster in parts of the RecA1/RecA2 domains and C-terminal region, which sit behind residue Q452 (Fig. 4a). A nonsense mutation at codon 452 created a truncated XPD, leading to severe XP for carriers as described in our report. XPD interacts with the four subunit proteins of the TFIIH complex (XPB, p62, p44 and MAT1; Fig. 4b). TFIIH forms a complex structure with these multiple proteins, wrapping around XPD to form a cradle-like structure, suggesting that XPD is strictly regulated. The p62 protein interacts with XPD at a number of different regions: (i) the RecA2 domain, (ii) the DNA-binding cavity and (iii) the cleft between the two RecA-like domains; these interactions regulate the binding and unwinding of DNA. XPB contacts XPD to play a role in the initial step of DNA binding and may regulate substrate binding by XPD at the ARCH domain of XPD. p44 has a relatively small interaction surface with XPD, and this leads to higher sensitivity to mutations at the XPD-p44 interface. Thus, numerous disease mutations at this interface were clustered near the helicase substrate-binding or active site of XPD. Therefore, the loss of the XPD C-terminus (from Q452 to the end of the protein) could disrupt the interactions with other components of the TFIIH complex, resulting in destabilizing the TFIIH core structure and diminishing/ablating XPD’s functions (Fig. 4b). This inevitably would lead to a breakdown of NER and the initiation of XP pathology.

In conclusion, we used an in-depth WES analysis to identify the genetic cause of XP in a family severely affected by the disease. Crucially, we describe a novel *ERCC2* variant that is responsible for the extreme disease phenotype witnessed in four siblings of this family. Genetic analysis, as presented in this study, is vital to guide the diagnosis, prevention and treatment of inherited conditions, including XP, in the future.

To investigate changes in the XPD stability caused by R683Q and Q452X (nonsense mutation), PoPMuSiC^15^ was applied to calculate the folding free energy (DDG, kcal/mol) of wild-type and mutant XPD structures. The analysis showed that R683Q destabilized the structure of XPD because of the positive DDG (Table 2). Most residues after Q452, if they are mutated, would disturb the overall structure of XPD because of the positive DDG (Table 2). This prediction is consistent with the clinical observations of XP patients.

**Table 2:** PoPMuSiC analysis of how single-site mutations of XPD alter the protein folding free energy (DDG) and stability.

Residue position	Wild-type residue	Mutant residue	Solvent accessibility (%)	ΔΔG (kcal/mol)	Stability prediction
461	L	V	0.83	1.4	Destabilizing
485	L	P	18.28	2.12	Destabilizing
541	S	R	15.22	1.5	Destabilizing
6161	R	P	4.44	0.39	Destabilizing
658	R	C	0.93	1.36	Destabilizing
681	D	N	0.00	1.39	Destabilizing
683	R	Q	6.48	0.83	Destabilizing
713	G	R	0.00	0.95	Destabilizing
717	A	G	0.00	1.63	Destabilizing
722	R	W	15.86	1.01	Destabilizing
725	A	P	6.62	1.62	Destabilizing
